# Exploring the protective potential of bilobalide against amyloid beta peptide 1‐42‐induced toxicity in BE(2)‐M17 cells: insights via cell viability and cellular energetics

**DOI:** 10.1002/alz.092076

**Published:** 2025-01-09

**Authors:** Manas Chakraborty, Kunal Dhiman, Veer Gupta

**Affiliations:** ^1^ Deakin University, Waurn Ponds, VIC Australia; ^2^ Deakin University, Highton, VIC Australia; ^3^ Deakin University, Geelong, VIC Australia

## Abstract

**Background:**

Reliable treatment approaches for addressing early cognitive impairment and Alzheimer’s disease (AD) are currently lacking. Given the multifactorial nature of AD, therapeutic strategies need to focus on disease‐specific biochemical pathways. Given the significance of metabolic pathways in cognitive impairment, it is essential to investigate alternative disease modifiers capable of targeting multiple metabolic pathways, such as phytochemicals. This research project aims to identify alterations in bioenergetics and viability resulting from amyloid pathology in cell models. Additionally, it will assess the therapeutic potential of a of bilobalide a biologically active terpenoid extracted from *Ginkgo biloba*. in alleviating these changes.

**Method:**

The Neuroblastoma cell line BE(2)‐M17 cells were utilized to establish an in vitro model of Alzheimer’s disease (AD). These cells underwent differentiation into mature human neurons through treatment with 10 µM retinoic acid. Prior to treatment with amyloid beta 1‐42 (20 µM) for 24 hours, the cells were pre‐treated with varying doses of bilobalide (1 µM, 5 µM, 10 µM, 15 µM, and 20 µM) for 24 hours. To assess the impact of bilobalide pretreatments on cell viability, a Cell Titer Glo assay was performed. The oxygen consumption rate, Total Reactive Oxygen Species (ROS), and 2',7'‐dichlorofluorescin diacetate (DCFDA) assays were conducted to evaluate cellular responses. Additionally, Seahorse analysis was employed to further investigate the oxygen consumption rate.

**Result:**

The successful differentiation of the BE(2)‐M17 cells has been achieved and confirmed through morphological changes. The cell viability results indicate a substantial impact of bilobalide in mitigating the amylopathic changes induced by the amyloid beta insult. While the cell energetics evaluation is still underway, the preliminary results from the pilot study strongly suggest a significant impact of bilobalide in alleviating the amylopathic changes induced by the amyloid beta insult

**Conclusion:**

The observed amelioration of amylopathic changes points towards the potential therapeutic effect of bilobalide in the context of Alzheimer’s disease. These initial findings provide promising indications of the potential effectiveness of bilobalide in influencing cellular energetics in the context of Alzheimer’s disease.

